# Development and characterization of microsatellite markers in *Gaultheria pumila* Lf. (Ericaceae)

**DOI:** 10.1186/s40659-018-0193-4

**Published:** 2018-11-01

**Authors:** Rolando Garcia-Gonzales, José Pico-Mendoza, Karla Quiroz, Basilio Carrasco, Pablo Cáceres, Borys Chong-Perez, Hugo Pino, Marjorie Seiltgens, Eglis Greck, Peter D. S. Caligari

**Affiliations:** 1 Sociedad de Investigación y Servicios, BioTECNOS Ltda, 49 ½ Oriente, 2385, Parque del Sol, Talca, Chile; 2 Facultad de Ingeniería Agronómica, Universidad Técnica de Manabí, Portoviejo, Manabí Ecuador; 3 Centro de Biotecnología de los Recursos Naturales, Departamento de Ciencias Forestales, Universidad Católica del Maule, Campus San Miguel, Av. San Miguel 3605, Casilla 617, Talca, Chile; 4 Facultad de Ciencias Agrarias, Universidad de Talca, Talca, Chile; 5 Facultad de Agronomía, Pontificia Universidad Católica de Chile, Avenida Vicuña Mackenna 4860, Macul, Santiago de Chile, Chile; 6 Verdant BioScience Pte. Ltda, 18, Duxton Hill, Singapore, 089601 Singapore

**Keywords:** Microsatellite, Pyrosequencing, Chilean berry, *Gaultheria pumila*

## Abstract

**Background:**

Polymorphic microsatellite markers were developed for *Gaultheria pumila* (Ericaceae) to evaluate genetic diversity and population structure within its native range in Chile. This is a very important Ericaceae endemic to Chile with a large commercial potential. Its resistance to different abiotic conditions makes it a valuable target for genetic improvement.

**Results:**

Ten polymorphic simple sequence repeat (SSR) loci were isolated from *Gaultheria pumila* using new-generation 454 FLX Titanium pyrosequencing technology. The mean number of alleles per locus ranged from 2 to 4. Observed and expected heterozygosity ranged from 0.00 to 1.0 and 0.00 to 0.64, respectively.

**Conclusions:**

From 10 SSR markers developed for *G. pumila*, 9 markers are promising candidates for analyzing genetic variation within or between natural populations of *G. pumila* and other species from the same genus.

## Background

The *Gaultheria* genus belongs to the Ericaceae family and is adapted to tropical and temperate conditions. In Chile, all of these species are commonly known as “chaura” or “mutilla del zorro” [1]. *Gaultheria pumila* is one of the most important species of this genus in Chile, regarding population size. In the wild, it is considered as a primary colonizing plant [2], capable of associating with symbiotic mycorrhizas [3]. The species shows a high degree of phenotypic diversity and even a botanic variety has been identified (*Gaultheria pumila* var. leucocarpa). It is an interesting species for domestication since it has shown a high level of phenotypic variability and high levels of polyphenols and anthocyanins [4, 5]. However, the lack of basic knowledge of agronomically important traits as well its genetic variability are serious limitations to its domestication process for its agronomic and commercial use. For this reason, the aim of this study was to develop a set of polymorphic microsatellite markers to be used as a tool to assess the diversity and genetic structure of *G. pumila* and other species of the *Gaultheria* genus.

## Methods

In order to carry out complete sequencing, four samples of *G. pumila* were collected in two places. The Villarrica volcano in Región de la Araucanía, Chile, S: 39°21′570, W: 71′57′865 (El Playón sector), and near the Puyehue volcano, in Región de Los Lagos, Chile, S: 40°41′484, W: 72°32′719 (Orilla del Río sector). All the samples from the protected natural areas were collected under the authorization of the regulatory Chilean Agency, Corporación Nacional Forestal (CONAF).

The samples for sequencing were selected according to fruit color: red, pink and white. Total genomic DNA of *G. pumila* was extracted from young leaves with a modified CTAB (cetyltrimethylammonium bromide) method [6]. The quality and quantity of isolated DNA was determined using a Nanodrop and agarose gel electrophoresis. The library preparation and shotgun pyrosequencing of 5 μg DNA aliquot on a 454 GS-FLX instrument (Roche Applied Science, MACROGEN, Ltd. Seoul, South Korea) was prepared with the kit of Titanium Pico Titer Plate (Roche Diagnostics), following the manufacturer’s protocols.

A total of 164,000 sequences were read, with an average length of 417 bp, which were generated by three independent runs. The sequences were assembled using the software GS De Novo Assembler v2.9 (http://gensoft.pasteur.fr/docs/454_DataAnalysis/2.9/USM-00058.09_454SeqSys_SWManual-v2.9_PartC.pdf).

The MSATCOMANDER (http://code.google.com/p/msatcommander/) software for locating microsatellites was used. MSATCOMMANDER is a program written to locate microsatellite repeats within fasta-formatted sequences or consensus files. MSATCOMMANDER will search for all di-, tri-, tetra-, penta, and hexa-nucleotide repeats. The search parameters were: mononucleotide repeat length: 10, dinucleotide repeat length: 6, trinucleotide repeat length: 4, tetranucleotide repeat length: 4, pentanucleotide repeat length: 4, and hexanucleotide repeat length: 4.

The primers were designed using the software PRIMER 3 [7]. The PCR fragment amplification and validation of selected SSRs were performed following the method developed by [8] which uses three primers: a forward primer with an M13 (-21) tail at its 5′ end, a normal reverse primer and the universal M13 (-21) primer labeled with either 6-FAM, VIC, PET or NED fluorochromes. The microsatellite information and GenBank accession numbers are listed in Table 1. PCR reactions were performed in a 20 μL reaction mixture with 10 ng templates DNA, 0.15 mM of each dNTP; 1× Taq polymerase reaction buffer; 1.5 mM MgCl_2_; 0.025 μM forward primer; 0.1 μM reverse primer; 0.1 μM M13 primer and 0.35 U Taq DNA polymerase.

**Table 1:** Characteristics of 10 microsatellite loci and primer pair’s development for *G. pumila*

Locus	Primer sequences (5′–3′)	Products size	Repeat motif	size (bp)	Dye	Ta (°C)	GenBank
GP.7	F: CGCATTCACTCACCCTCTCA	309	(ACTC)^4	360	FAM	52.1	KX719822
	R: TGGTTGGTGAAGGCTTTGGA						
GP.9	F: ACCCGCTCTAGATCCTCTCT	321	(T)^10	378–380	FAM	60.5	KX719823
	R: AGGGGGAGTAATCAAGCCTCT						
GP.10	F: GGGTACGGCGTAGTGGTAAT	269	(AT)^8	323–327	VIC	60.5	KX719824
	R: TCGTACAAAACCGCCCTCAA						
GP.12	F: AGGATTATAGAGAGCCAGGTGGA	138	(CTT)^4	198–201	NED	52.1	KX719825
	R:CAGAAGACGAAATCGAAGCCG						
GP.13	F: AGAGTAAGAGCTCTCTTCCGA	248	(ATT)^4	161–278	NED	52.1	KX719826
	R: GCAGACTCGAATCGGCAGTA						
GP.14	F: GCATAGCCCGGTTGTCAAAC	174	(ACGC)^4	196–228	PET	52.1	KX719827
	R: ACCGAAAGATCCGACCATCG						
GP.15	F: GGGCTGCTGCTCAATCAATT	287	(T)^10	347–351	VIC	52.1	KX719828
	R: ACCCGCTTCAAGTCATGATGA						
GP.16	F: GCTATTTCTAGGGCCGGACC	218	(AT)^6	281–283	FAM	52.1	KX719829
	R: GCACAATACATAGATTCTGGATCGA						
GP.17	F: GAGAGAAATCCACCAGGGCA	145	(T)^10	202–205	VIC	60.5	KX719830
	R: CAAGCGGACGACGTATACGA						
GP.18	F: ACCGAAAGATCCGACCATCG	174	(GCGT)^4	192–228	PET	52.1	KX719831
	R: GCATAGCCCGGTTGTCAAAC						

For each locus, the name, primer sequence, products size, repeat motif, allele size range (bp), fluorescent dye, annealing temperature (Ta) and GenBank accession numbers

PCR amplifications were performed in an Applied Biosystems Veriti (Life Technologies), under the following conditions: initial denaturation at 94 °C for 5 min; 30 cycles of 30 s (s) at 94 °C, annealing temperature specific to each primer pair for 45 s, extension at 72 °C for 45 s, followed by 8 cycles of 30 s at 94 °C, annealing at 53 °C for 45 s, extension at 72 °C for 45 s and a final extension at 72 °C for 30 min. The annealing temperature (°C) requirements of primers are specified in Table 1.

PCR products were resolved as following: 2 μL of PCR products were mixed with 10 μL HiDi formamide (Applied Biosystems) and 0.2 μL GeneScan 500LIZ size standard (Life Technologies, Foster City, CA) and separated by capillary electrophoresis on an ABI 3130xl Prism Genetic Analyzer with POP-7 polymer (Life Technologies, Foster City, CA) in the Genetic Resources Unit, La Platina-INIA, Chile. Allele sizes were automatically calculated with GeneMapper software v4.0 and manually checked.

Three populations of *G. pumila* were selected to evaluate the variability in the isolated loci: Región de la Araucanía, (Villarrica volcano, n = 10); Región de los Lagos, (Puyehue volcano, n = 10); Región de Magallanes y la Antártica chilena (Punta Arenas, n = 10). Genetic parameters such as observed number of alleles (*Na*) and observed and expected heterozygosity (*Ho*–*He*) were estimated with PopGene [9]. The inbreeding coefficient (*F*_*IS*_) [10] was determined using Genetix [11]. Deviation from the Hardy–Weinberg equilibrium was determined with GENEPOP v 4.2 [12] (Table 2). The presence of null alleles was checked using MICRO-CHECKER version 2.2.3 [13].

**Table 2:** Estimated genetic parameters and Hardy–Weinberg test results in 10 microsatellites loci in three wild populations of *G. pumila*

Locus	Puyehue volcano (n = 10)				Villarrica volcano (n = 10)				P. Arenas (n = 10)				Cross-amplification	
	Na	Ho	He	Fis	Na	Ho	He	Fis	Na	Ho	He	Fis	*G. mucronata*	*G. caespitosa*
GP.7	1	0.00	0.00	NA	1	0.00	0.00	NA	1	0.00	0.00	NA	−	−
GP.9	1	0.00	0.00	− 0.24	2	0.20	0.19*	− 0.34	2	0.56	0.42	− 0.06*	−	+
GP.10	2	0.30	0.27	− 0.27	3	0.67	0.50*	− 0.10	2	0.22	0.21	− 0.27*	−	−
GP.12	2	0.00	0.18*	1.00	2	0.00	0.19*	1.00	2	0.00	0.19*	1.00*	−	−
GP.13	2	1.00	0.53*	− 0.46	4	0.60	0.59*	− 1.00	2	1.00	0.53*	− 0.46	−	+
GP.14	2	0.10	0.10	− 0.00	1	0.00	0.00	0.00	1	0.00	0.00	0.00	+	+
GP.15	3	0.70	0.67	0.45	3	0.22	0.52	0.13	3	0.40	0.58	0.22*	+	+
GP.16	1	0.00	0.00	1	2	0.00	0.34	1.00	2	0.00	0.51*	− 1.00	+	+
GP.17	2	1.00	0.53*	0.70	2	1.00	0.53	− 1.00	2	1.00	0.53*	0.49	+	−
GP.18	2	0.10	0.10	0.13	3	0.10	0.28*	− 0.03	1	0.00	0.00	0.10	+	+

+, successful PCR amplification; −, unsuccessful PCR amplification

*Na* number of alleles, *Ho* observed heterozygosity, *He* expected heterozygosity, *Fis* fixation index

*Significant deviation from Hardy–Weinberg equilibrium (P < 0.05)

In addition, cross-amplification was tested in two other species, *Gaultheria mucronata* and *Gaultheria caespitosa*. PCR reactions and electrophoresis were performed according to the conditions described above.

## Results

Primers were considered successful when one clear distinct band was detected on 2% agarose gel. Primers were designed with flanking repeat lengths of at least 10, 6 and 4 for mononucleotides, dinucleotides, and higher core repeats, respectively. Mononuclotide-containing repeats were the most frequent (59%), followed by dinucleotides (23%), trinucleotides (12%), tetranucleotides (3%) and pentanucleotides (2%).

Seventeen SSRs were tested and they included primers for four mononucleotides, five dinucleotides, four trinucleotides, three tetranucleotides, and one pentanucleotide. The first test consisted of selecting the best SSRs that showed amplification in a 2% agarose gel after electrophoresis. Ten SSRs were selected for further development and analysis. From these microsatellite loci, nine were variable and polymorphic between populations.

The microsatellite GP.7 was inconsistent in different tested condition, and it was not included further. The mean number of alleles per locus ranged from 1 to 4, with an average of 1.96. Observed and expected heterozygosity ranged from 0.00 to 1.0 and 0.00 to 0.67, respectively. In addition, three microsatellites loci GP.12, GP.13, GP.17 exhibited significant deviation from Hardy–Weinberg equilibrium, for the Puyehue population. In the Villarrica population GP.14 was monomorphic, while the loci GP.9, GP.10, GP. 12, GP.13 and GP.18 had no significant deviations. In Punta Arenas, four loci (GP.9, GP.10, GP.12 and GP.15) did not show significant deviations from HWE. Such deviations can be due to the small sample size, selfing, or to the substructure of the populations. Additionally, after visualization of the PCR products for each loci by gel electrophoresis, it was found that out of 10 SSR loci, 5 showed cross amplification in *G. mucronata* (GP.14, GP.15, GP.16, GP.17 and GP.18) and 6 *G. caespitosa* (GP.9, GP.13, GP.14, GP.15, GP.16 and GP.18) (Table 2). This suggests that the markers could be useful in carrying out studies of genetic diversity in other *Gaultheria* species.

## Conclusions

Nine microsatellites primer pairs were developed and proved to be polymorphic in three populations of *G. pumila*. These SSRs will be useful for future studies of population structure and assessment of diversity in this species. Also, these findings provide a basis for starting a domestication program for this species, as well as other related species in the Ericaceae family. Further research will be needed to find new SSR microsatellite markers and test them among *G. pumila* and other species from this genus.

## Abbreviations

CONAF: Corporación Nacional Forestal
CTAB: cetyltrimethylammonium bromide
SSR: simple sequence repeat
PCR: polymerase chain reaction
